# Subregional variation in cover and diversity of hard coral (*Scleractinia*) in the Western Province, Solomon Islands following an unprecedented global bleaching event

**DOI:** 10.1371/journal.pone.0242153

**Published:** 2020-11-11

**Authors:** Danielle Denley, Anna Metaxas, Robert Scheibling

**Affiliations:** 1 Department of Oceanography, Dalhousie University, Halifax, Nova Scotia, Canada; 2 Department of Biology, Dalhousie University, Halifax, Nova Scotia, Canada; California Academy of Sciences, UNITED STATES

## Abstract

Coral reefs are critically important marine ecosystems that are threatened worldwide by cumulative impacts of global climate change and local stressors. The Solomon Islands comprise the southwestern boundary of the Coral Triangle, the global center of coral diversity located in the Indo-Pacific, and represent a bright spot of comparatively healthy coral reef ecosystems. However, reports on the status of coral reefs in the Solomon Islands are based on monitoring conducted at 5 stations in 2003–2004 and 2006–2007, with no information on how corals in this region have responded to more recent global bleaching events and other local stressors. In this study, we compare reef condition (substrate composition) and function (taxonomic and morphological diversity of hard corals) among 15 reefs surveyed in the Western Province, Solomon Islands that span a range of local disturbance and conservation histories. Overall, we found high cover of live hard coral (15–64%) and diverse coral assemblages despite an unprecedented 36-month global bleaching event in the three years leading up to our surveys in 2018. However, there was significant variation in coral cover and diversity across the 15 reefs surveyed, suggesting that impacts of global disturbance events are moderated at smaller scales by local anthropogenic factors (fisheries extraction, land-use impacts, marine management) and environmental (hydrodynamics) conditions. Our study provides evidence that relatively healthy reefs persist at some locations in the Solomon Islands and that local stewardship practices have the potential to impact reef condition at subregional scales. As coral reef conservation becomes increasingly urgent in the face of escalating cumulative threats, prioritising sites for management efforts is critical. Based on our findings and the high dependency of Solomon Islanders on coral reef ecosystem services, we advocate that the Western Province, Solomon Islands be considered of high conservation priority.

## Introduction

Coral reefs are diverse and highly productive marine ecosystems that are increasingly threatened by human activities and climate change [1–3]. Local stressors such as overfishing, sedimentation and nutrient pollution, occurring against a backdrop of global increases in ocean temperature, ocean acidification and the intensity of tropical cyclones (hurricanes) have led to dramatic reductions in reef health and the effective loss of 19% of the world’s coral reefs [4]. The Indo-Pacific contains 76% of the world’s reef-building coral species and is recognized as the global center of coral diversity [5]. Many coastal ecosystems in the Indo-Pacific are considered to be ecologically resilient due to high levels of biodiversity and rapid rates of growth and recovery [2]. However, loss of coral in this region may be occurring faster than expected, with coral cover declining by an annual average of 2% between 1997 and 2003 [6].

The Solomon Islands is an archipelago of 992 islands divided into 9 provinces that comprise the southwestern boundary of the Coral Triangle in the Indo-Pacific. Coral reef monitoring in the Solomon Islands was minimal prior to 2003 when permanent monitoring stations (4 in the Western Province and 1 in Isabel Province) were established by the Solomon Islands Coral Reef Monitoring Network [7, 8]. These stations were monitored in 2003–2004 and again in 2006–2007, and the results form the basis of reports on the status of coral reefs in the Solomon Islands (ReefBase, reefbase.org). The Solomon Islands have represented a “bright spot” for coral reefs, where reef ecosystems are in significantly better condition than expected based on regional environmental conditions and socioeconomic drivers [9]. The relatively healthy condition of coral reefs in the Solomon Islands is mainly the result of reduced impacts of global drivers such as warming, augmented by local community-based management of marine resources implemented through customary marine tenure and the establishment of Locally Managed Marine Areas (LMMAs) [10, 11]. There are 113 LMMAs containing an estimated 155 no-take zones in the Solomon Islands [8]. The largest LMMA, with a contiguous 13 km no-take zone, is on Tetepare Island and is managed by the Tetepare Descendants’ Association.

The Solomon Islands have the highest rate of population growth in the Coral Triangle and are ranked as being among the island nations with the greatest reliance on reefs and the lowest capacity to adapt to reef degradation and loss [2, 10]. Consequently, protecting Solomon Island reefs from combined increases in both global and local anthropogenic stressors is of critical importance to avoid substantial ecological, social and economic impacts [2]. Despite recent progress, such as the establishment of the Tetepare marine protected area in 2003, barriers to conservation still exist. Among them is the lack of an information database on the status of coral reefs in the Solomon Islands that can be used to quantify changes in reef health and evaluate the effectiveness of various management strategies [12].

In this study, we surveyed reefs at 13 sites spanning 4 islands in the Western Province, Solomon Islands following the unprecedented 36-month global bleaching event in 2014–2017 [13]. Bleaching events can alter the community composition of coral reefs by changing the relative abundance of coral taxa or morphologies, as well as that of other taxa, based on the susceptibility of corals to bleaching-induced mortality [1]. We compare metrics of reef condition and function (substrate composition, taxonomic and morphological diversity of hard corals) among reefs that span a range of local disturbance and conservation histories. Although local environmental conditions offer minimal resistance to global-scale impacts such as severe bleaching events [14], reducing local stressors through the implementation of LMMAs to protect fish stocks and reduce nutrient levels may enhance the capacity of reefs to recover from regional disturbances such cyclones and bleaching [15]. Our results provide critical baseline data on the status of these coral reefs in the Solomon Islands following prolonged global bleaching and elucidate the role of local conditions in mitigating impacts of large-scale disturbance events.

## Methods

### Data collection

**Fig 1:**
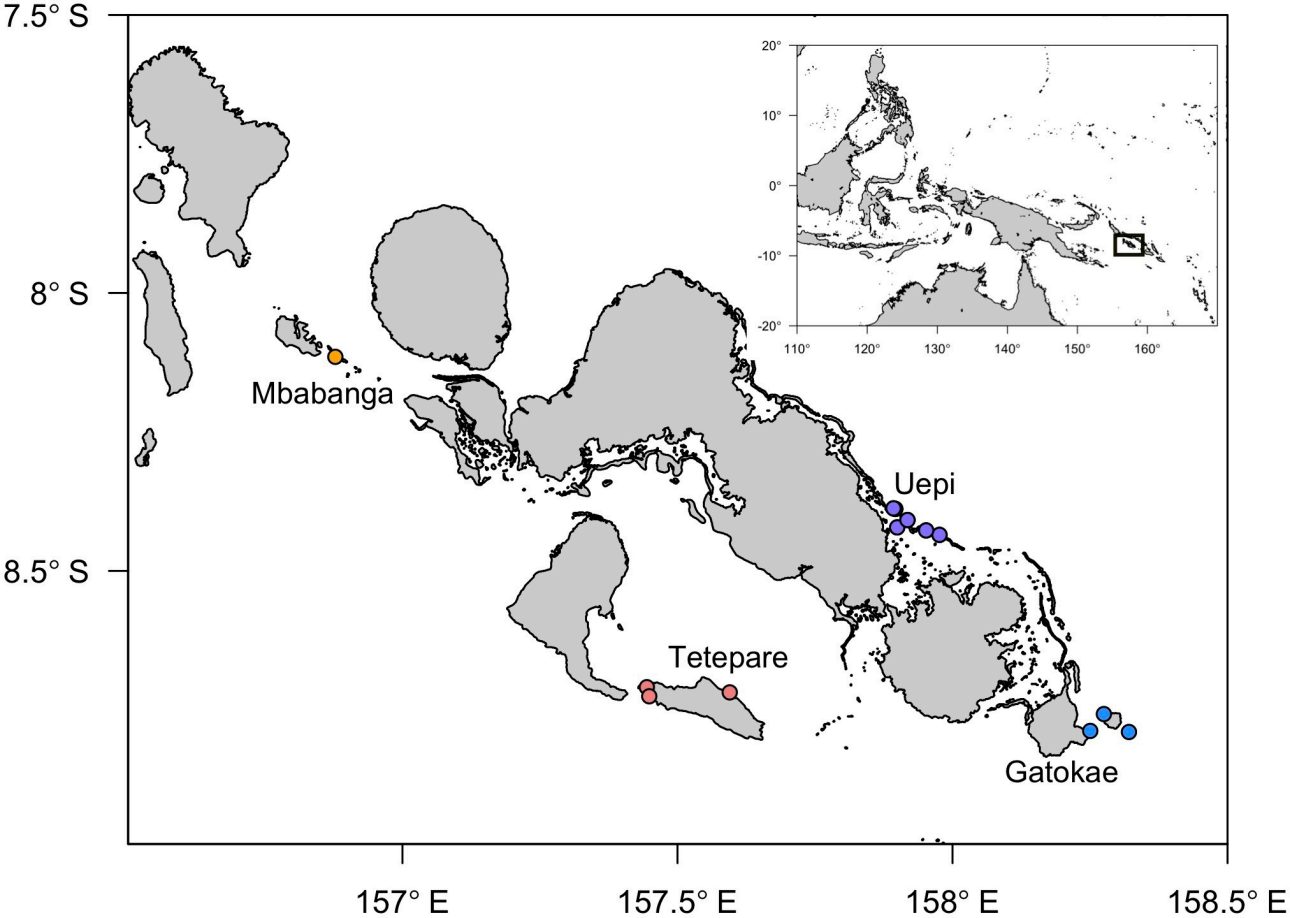
Locations of sites where coral reefs were surveyed in the Western Province, Solomon Islands. Sites were located among 4 islands: Mbabanga (orange dots), Tetepare (pink dots), Uepi (purple dots), Gatokae (blue dots). Site names, in order of geographic location from northwest to southeast, are: Mbabanga (MbaS, MbaD); Tetepare: Site 4 (Sit), Lagoon (Lag), Serunama (Ser); Uepi: Liplipate (Lip), Binusa (Bin), Landoro Gardens (Lan), Roma (RomS, RomD), Uepi Point (Uep), Secret Spot (Sec); Gatokae: Male Male (Mal), Mbula (Mbu), Kitcha (Kit). Map generated using the PBSmapping package in R (Jon T. Schnute, Nicholas Boers and Rowan Haigh (2019). PBSmapping: Mapping Fisheries Data and Spatial Analysis Tools. R package version 2.72.1. https://CRAN.R-project.org/package=PBSmapping).

We surveyed coral reefs at 13 sites in the Western Province, Solomon Islands from 13 to 31 May 2018. Sites were located at 4 islands (Mbabanga, Tetepare, Uepi, Gatokae), ranging from Mbabanga in the northwest (08° 06’ 54” S, 156° 52’ 41” E) to Kitcha (on Gatokae) in the southeast (8° 47’ 23” S, 158° 19’ 15” E) (Fig 1, Table 1). Site selection was opportunistic, based on where dive operators were permitted to visit. A 120-m video transect was sampled at each site using scuba, except for Uepi Point and Male Male (60-m transects) and Landoro Gardens (93-m transect) where the reef was not extensive enough to accommodate the longer transects. Video transects followed a near-constant depth contour, and height above the coral was maintained at ~1.5 m using a plumb line weighted with a 3.8-cm diameter metal washer as a scaling element. Approximate depths of video transects ranged among sites from 2 to 10 m (Table 1). At 2 of the 13 sites, Mbabanga and Roma, two 120-m video transects were sampled, one at each of 5–7 m and 9–10 m depth, and 6–7 m and 9–10 m depth, respectively (Table 1). We also calculated degree heating weeks (DHW, °C wk), a metric that incorporates both the magnitude and duration of heat stress, for each of the 4 islands surveyed for 2013–2018 using satellite-derived sea surface temperature (SST) data acquired from NOAA Coral Reef Watch (2018). We compared SST and DHW for each island in each year to established thresholds for bleaching (31°C) and bleaching Alert Levels 1 (4 DHW) and 2 (DHW), respectively (S1 Fig).

**Table 1:** Details of 13 sites surveyed in the Western Province, Solomon Islands between May 13–31, 2018.

Island	Site (Abbr)	Location	Depth (m)	Transect	Disturbance history
Mbabanga	Mbabanga shallow (MbaS)	08° 06’ 53.7” S 156° 52’ 40.6” E	6–7	120-m	8.1 magnitude earthquake, Apr 2007 [35] Moderate damage to branching corals, a few dislodged and overturned corals, but no impact from resulting Tsunami (Hans Mergozzi, Managing Director at Sanbis Resort, *pers comm*) Bleaching event, Jan–May 2000 [12] Extensive bleaching of plate and staghorn (*Acropora*) corals Crown of Thorns Starfish (COTS) removal since 1999 No records kept but estimated removal of ~400 individuals in Mar–May 2018 (Hans Mergozzi, Managing Director at Sanbis Resort, *pers comm*) At nearby Imagination Island: Coral covered in algae and coral rubble in 2011 COTS eradication initiated October 2016, ~ 4,000 individuals removed in 2016–2018 (Gilli, assistant to JT Ward, Owner Manager, Imagination Island Coral Reef Resort, *pers comm*)
Mbabanga	Mbabanga deep (MbaD)	08° 06’ 53.7” S 156° 52’ 40.6” E	9–10	120-m	
Tetepare	Site 4 (Sit)	08° 42’ 33.0” S 157° 26’ 39.5” E	2–4	120-m	No take MPA established in 2003 (Site 4 and Lagoon only)^1^ Low severity bleaching in 2009 (Site 4 and Lagoon only)^2^ Tsunami, Jan 2010^3^ Some damage recorded on reefs^4^, upturned individual corals but no extensive areas of dead or damaged corals
Tetepare	Lagoon (Lag)	08° 43’ 31.8” S 157° 26’ 55.3” E	2–4	120-m	
Tetepare	Serunama (Ser)	08° 43’ 06.6” S 157° 35’ 42.1” E	2–4	120-m	
Uepi	Uepi Point (Uep)	08° 25’ 37.8” S 157° 57’ 7.4” E	5.5	60-m	Bleaching event, Jan–May 2000 [12] 20% of coral bleached, up to 50% at shallow sites Over-fishing [37] Increased terrestrial and sediment input due to land-use changes, 1995–2005 [37] Bleaching of tabular and digitate *Acropora*, 2005 (Uepi Point and Secret Spot only) [37] Minimal COTS and associated coral damage as of 2005 (Uepi Point and Secret Spot only)^5^ High mortality of diseased corals, 2004 (Uepi Point only)^6^ Patchy occurrence of COTS; not found at all reefs. No records but estimated removal of 1000s of individuals (Grant Kelly, owner and manager Uepi Island Resort, *pers comm*) Extensive bleaching in 2017 at Binusa only, deeper corals (~20 m) more affected than shallow corals (Jill Kelly *pers comm*) Some bleaching since December 2017 (Grant Kelly, *pers comm*) COTS outbreak 2017 at Binusa only (Jill Kelly *pers comm*) Bleaching in 1992 (100% of corals) at Landoro Gardens only (Jason Kelly *pers comm*)
Uepi	Liplipate (Lip)		7–10	120-m	
Uepi	Binusa (Bin)		5–8	120-m	
Uepi	Landoro Gardens (Lan)		5–7	93-m	
Uepi	Roma shallow (RomS)		5–7	120-m	
Uepi	Roma deep (RomD)		9–10	120-m	
Uepi	Secret Spot (Sec)		5–7	120-m	
Gatokae	Male Male (Mal)	08° 47’ 16.1” S 158° 15’ 01.6” E	4–5	60-m	6.2 magnitude earthquake aftershock, April 2007^7^ Commercial logging (Bulo Enterprises) until 2004 (Mbula only)^8^ Logging ongoing for ~50 yr (Stewart Steven, dive guide at Wilderness Lodge, *pers comm*) COTS outbreak ~2013–2018 at Male only (Stewart Steven, dive guide at Wilderness Lodge, *pers comm*) 10 COTS observed at Male in this study (2 along transect)
Gatokae	Mbula (Mbu)	08° 45’ 26.9” S 158° 16’ 30.7” E	5–6	120-m	
Gatokae	Kitcha (Kit)	08° 47’ 23.0” S 158° 19’ 14.9” E	5–7	120-m	

COTS is Crown-of-Thorns Starfish. Locations of sites on Uepi other than Uepi Point have been omitted at the request of the local community.

1Atlas of Marine Protection. An initiative of Marine Conservation Institute. [cited 2019 Sep 24]. Available from: http://www.mpatlas.org/mpa/sites/14514/.

2reefbase.org.

3The Associated Press. Destructive tsunami crashes over Solomon Islands. 2010 Jan 4. In: The San Diego Union Tribune [Internet]. San Diego, California [cited 25 September 2019]. Available from: https://www.sandiegouniontribune.com/sdut-destructive-tsunami-crashes-over-solomon-islands-2010jan04-story.html.

4Tetepare Visitor Guidebook; 2012.

5Kinch J, Mesia P, Kere N, Manioli J, Bulehite K. Socioeconomic baseline study: Eastern Marovo Lagoon, Solomon Islands. Apia, Samoa: SPREP;2006: IWP-Pacific Technical report, ISSN 1818- 5614, no.35.

6Green A, Lokani R, Atu W, Ramohia P, Thomas P, Almany J, editors. Solomon Islands Marine Assessment: Technical report of survey conducted May 13 to June 17, 2004. TNC Pacific Island Countries Report No. 1/06.

7Pararas-Carayannis G. Disaster Pages. 2007 [cited 24 September 2019]. Available from: http://www.drgeorgepc.com/Tsunami2007Solomons.html.

8Radio New Zealand. Solomons Marovo logging to be probed. 2004 April 15 [cited 24 September 2019]. In: Radio New Zealand [Internet]. Wellington, New Zealand. Available from: https://www.rnz.co.nz/international/pacific-news/148169/solomons-marovo-logging-to-be-probe.

### Ethics statement

No physical samples were collected for this study and no permits were required. Dive sites were accessed with a dive guide except at Mbabanga where they were located within 50 m from the resort. Access to the sites at Tetepare was granted by the Tetepare Descendants’ Association on Tetepare island and was accompanied by rangers.

### Video analysis

Frame-grabs (~0.5 m2) were sampled from each video transect at 20 to 40-s intervals, giving 16–52 video quadrats per transect, depending on transect length and swimming speed of the recorder (mean ± SD swim speed across all transects = 0.2 ± 0.06 m s-1). This provided sufficient sampling resolution to characterise reef assemblages at each site, as indicated by sample-based rarefaction curves (S2 Fig), while preventing image overlap.

**Fig 2:**
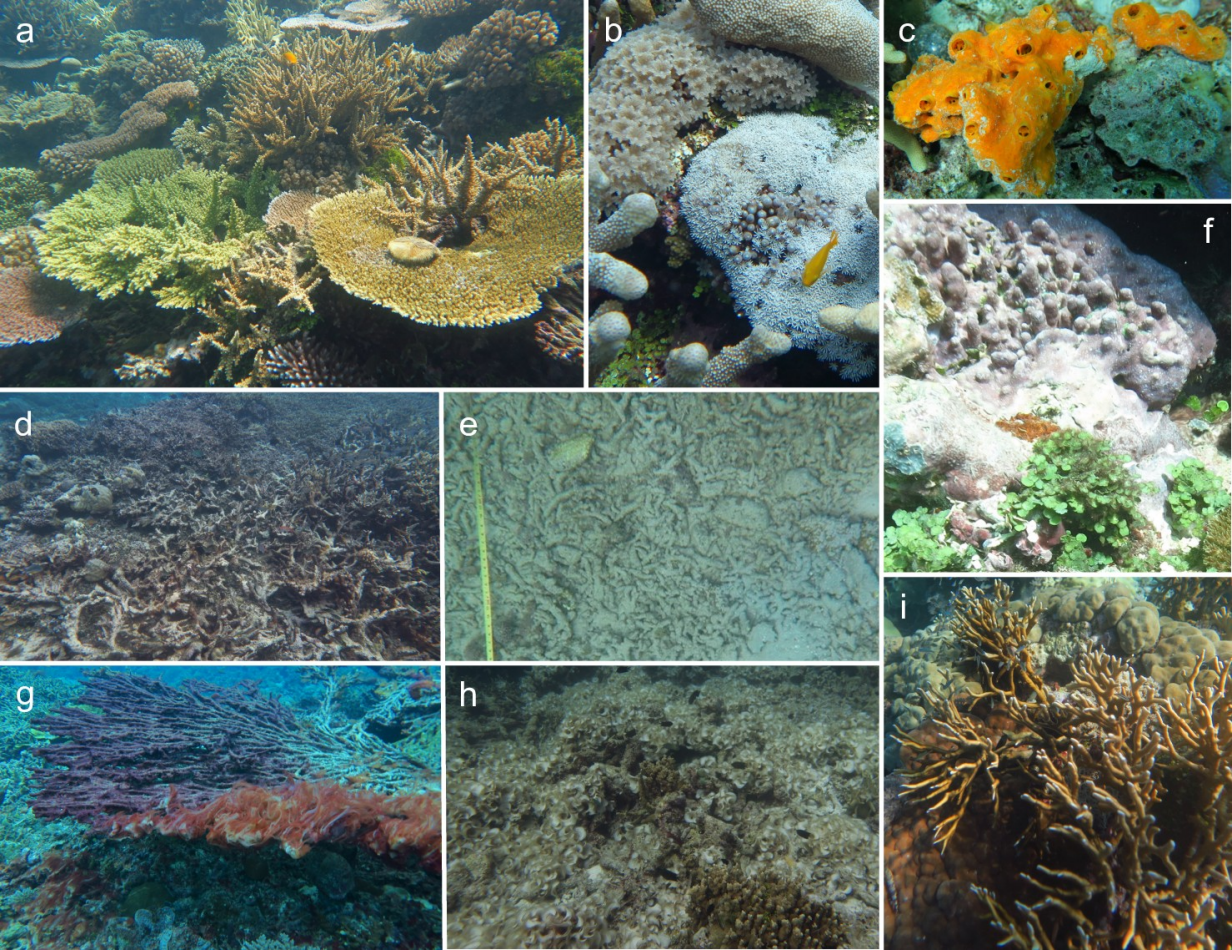
Predominant substrate types at 13 sites spanning 4 islands in the Western Province, Solomon Islands. a) live hard coral (Ser, Tetepare), b) soft coral (Mbu, Gatokae), c) sponge (Mba, Mbabanga), d) dead coral (Mal, Gatokae), e) coral rubble (Lag, Tetepare), f) coralline algae (Lip, Uepi), g) non-coralline algae: filamentous red algae (Rom, Uepi), h) non-coralline algae: funnel weed (Mba, Mbabanga), i) Other: hydrocoral (Mbu, Gatokae). Other substrate categories not pictured included rock/bedrock and sand. See Fig 1 for site abbreviations, in parentheses.

Video quadrats were analysed for percent cover of substrate type using the multi-point tool in ImageJ. A grid of 112 points was overlaid on each image and the substrate type beneath each point was identified. Nine substrate categories were defined to encompass the predominant biotic and abiotic substrates: live hard coral, soft coral, sponge, dead coral, rock/bedrock, sand, coral rubble, coralline algae, non-coralline algae (Fig 2). An additional category “Other” was used to represent rarely occurring species (e.g. macroinvertebrates, hydrocorals) or substrate types beyond the scope of this study (e.g. terrestrial debris) (Fig 2). Points for which substrate type could not be identified due to poor resolution (e.g. shadow, edge effects) were excluded from the calculation of percent cover (mean ± SD point exclusions per quadrat = 4.5 ± 3.5). Percent cover of each substrate type was averaged across all quadrats for each transect (S1 Data).

**Fig 3:**
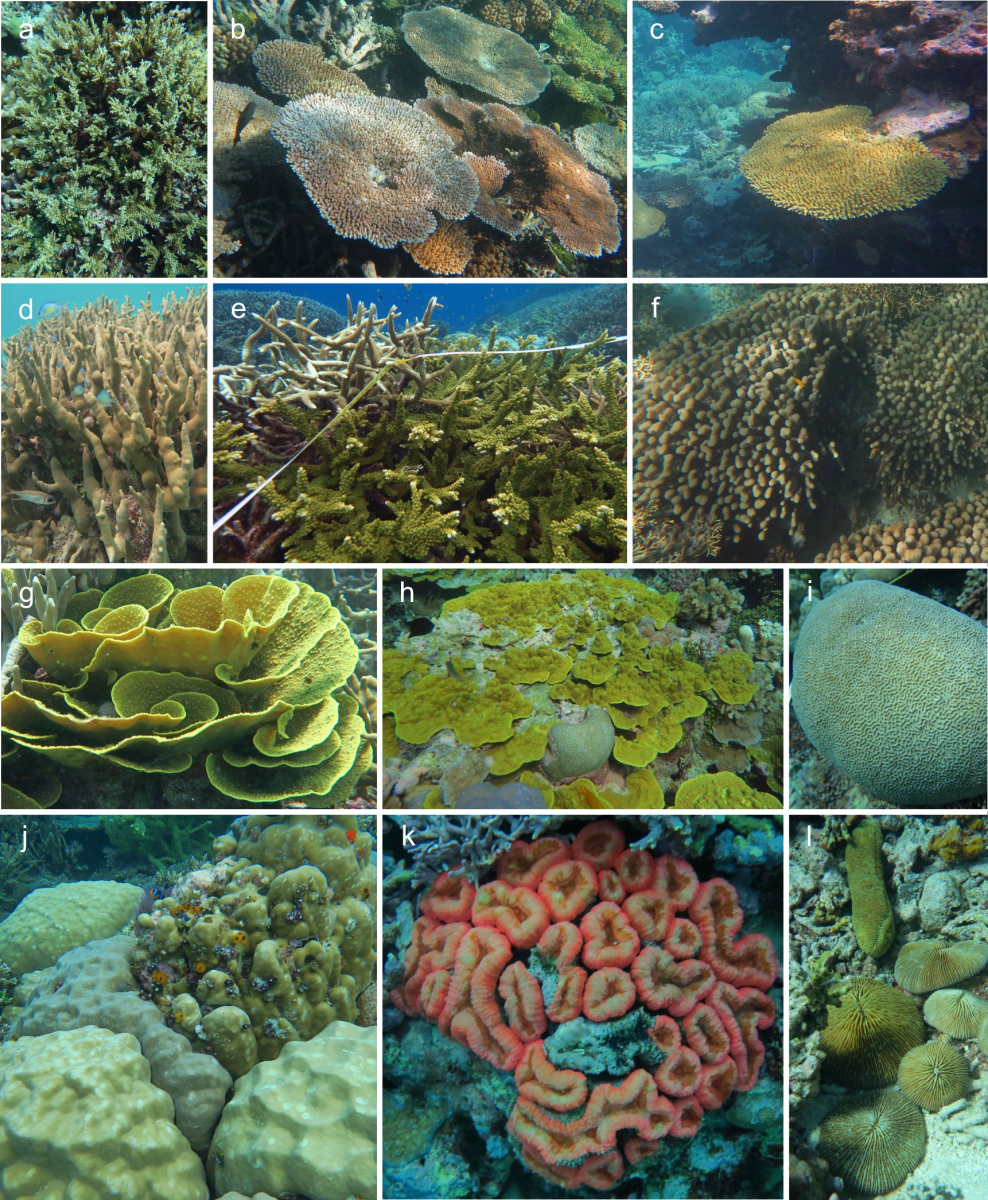
Examples of coral morphology categories and representative taxa described in Table 2. a) *Acropora* bottlebrush (Lan, Uepi), b) *Acropora* table (Ser, Tetepare), c) *Acropora* plate (Sec, Uepi), d) branching, *Porites* (Mba, Mbabanga), e) branching, *Acropora* (Kit, Gatokae), f) columnar, *Pavona* (Lag, Tetepare), g) foliaceous, *Turbinaria* (Mba, Mbabanga), h) laminar, *Porites* (Sec, Uepi), i) massive, *Platygyra* (Rom, Uepi), j) massive (foreground) and submassive (background), *Porites* (Kit, Gatokae), k) hemispherical, *Lobophyllia* (Rom, Uepi), l) free-living solitary, *Fungiidae* (Sec, Uepi). See Fig 1 for site abbreviations, in parentheses.

We identified the taxonomy (to genus) of all hard corals (*Scleractinia*, hereon referred to as corals) in each video quadrat. The absence of physical samples limited our ability to confidently resolve coral species and their specific life history strategies. Therefore, we also identified corals by morphology, which enabled us to somewhat distinguish among life histories within the same genus in some cases and provide a more complete representation of the structural complexity and functional diversity of the reefs we surveyed. The clonal nature of corals makes it difficult to identify individuals from images; therefore, taxonomy and morphology were recorded for each individual point overlaying live coral. We defined 23 morphological categories modified after Veron [16] and Edinger and Risk [17] (Table 2). Live corals were identified to genus using video and accompanying high resolution “context photos” from each site (Fig 3). Corals that could not be identified to genus were categorized as “unknown” and assigned a morphological category only (2.3% of cases, *n* = 20,481 points). The proportional abundance of each coral genus or morphological group was calculated for each transect as the number of points overlaying that taxon or morphology divided by the total number of points of coral (S2 Data).

**Table 2:** Categories used to quantify coral morphological composition at 13 sites in the Solomon Islands.

Coral morphology	Morphological description
*Acropora*, bottlebrush	Numerous short side branchlets projecting from a main branch
*Acropora*, digitate	Short non-divided branches like the fingers of a hand
*Acropora*, caespitose	Busy colonies with branches interlocking in three dimensions
*Acropora*, corymbose	Stout bushy colonies with horizontal anastomosing branches and short upright branchlets
*Acropora*, table	Flat colonies with one central leg (most are also corymbose)
*Acropora*, plate	Flat colonies attached to the substrate at one side (most are also corymbose)
Branching	Forming branches (includes arborescent)
Columnar	Forming columns
Foliaceous	Foliose, often forming whorls, mainly *Turbinaria*
Encrusting	Adhering to the substrate
Encrusting/branching	Corals with an encrusting base and branches
Encrusting/columnar	Corals with an encrusting base and columns
Encrusting/upgrowths	Corals with an encrusting base and short upgrowths
Laminar	Plate-like growth, often forming tiers
Laminar/branching	Corals with a laminar base and branches
Laminar/columnar	Corals with a laminar base and columns
Laminar/upgrowths	Corals with a laminar base and short upgrowths
Massive	Corals of all sizes that are solid and similar in size in all dimensions
Submassive	Multilobate or “lumpy” corals of all sizes
Hemispherical	Half-domed colonies, mainly *Lobophyllia*
Free-living colonial	Corals composed of many individuals that are not attached to the substrate
Free-living solitary	Corals composed of single individuals that are not attached to the substrate
Solitary	Corals composed of single individuals that may or may not be attached to the substrate, mainly *Cynarina*

Coral morphology categories were modified after [16, 17] and defined to encompass the predominant morphologies observed across all 13 sites (Fig 3).

### Statistical analysis

To examine differences in substrate cover (%) and taxonomic and morphological composition of coral assemblages (proportional abundance of each coral taxon and morphology) among sites we used non-metric multi-dimensional scaling (nMDS). Substrate cover (%) and proportional abundance data were arcsine square-root transformed prior to analysis and site ordination values were generated using the Bray-Curtis dissimilarity index.

To compare coral taxonomic richness and morphological complexity among sites we used sample-based rarefaction curves with video quadrats as samples and moment based estimation of confidence limits [18]. Rarefaction curves differ from corresponding species accumulation curves in that they are generated by repeated, randomized re-sampling of all pooled samples (video quadrats) without replacement, and therefore can be interpreted statistically. We chose to use a sample-based approach to account for spatial heterogeneity (patchiness) along transects, however, we rescaled our sample-based rarefaction curves to adjust for differences in coral density among sites by plotting the number of coral taxa or morphologies against the number of individuals (based on the average number of individuals per sample) after [19]. Because the clonal nature of corals makes it difficult to identify and count individuals, we considered the number of individuals to be the number of points overlaying live coral. Under this assumption, we plotted the number of coral taxa and morphologies against the number of individual points of live coral (based on the average number of points of live coral per sample). To examine taxonomic and morphological richness among sites at the same sampling effort, we compared the rarefied number of coral taxa and morphologies and the corresponding 84% confidence intervals for the minimum number of points of live coral sampled across all sites (genera: *n* = 407, morphologies: *n* = 411). We chose 84% confidence intervals to approximate a type I error rate of α < 0.05 [20], such that non-overlapping confidence intervals indicate significant differences in taxonomic and morphological richness among sites at *P* < 0.05. All analyses were performed using the *vegan* package in R [21, 22].

## Results

**Fig 4:**
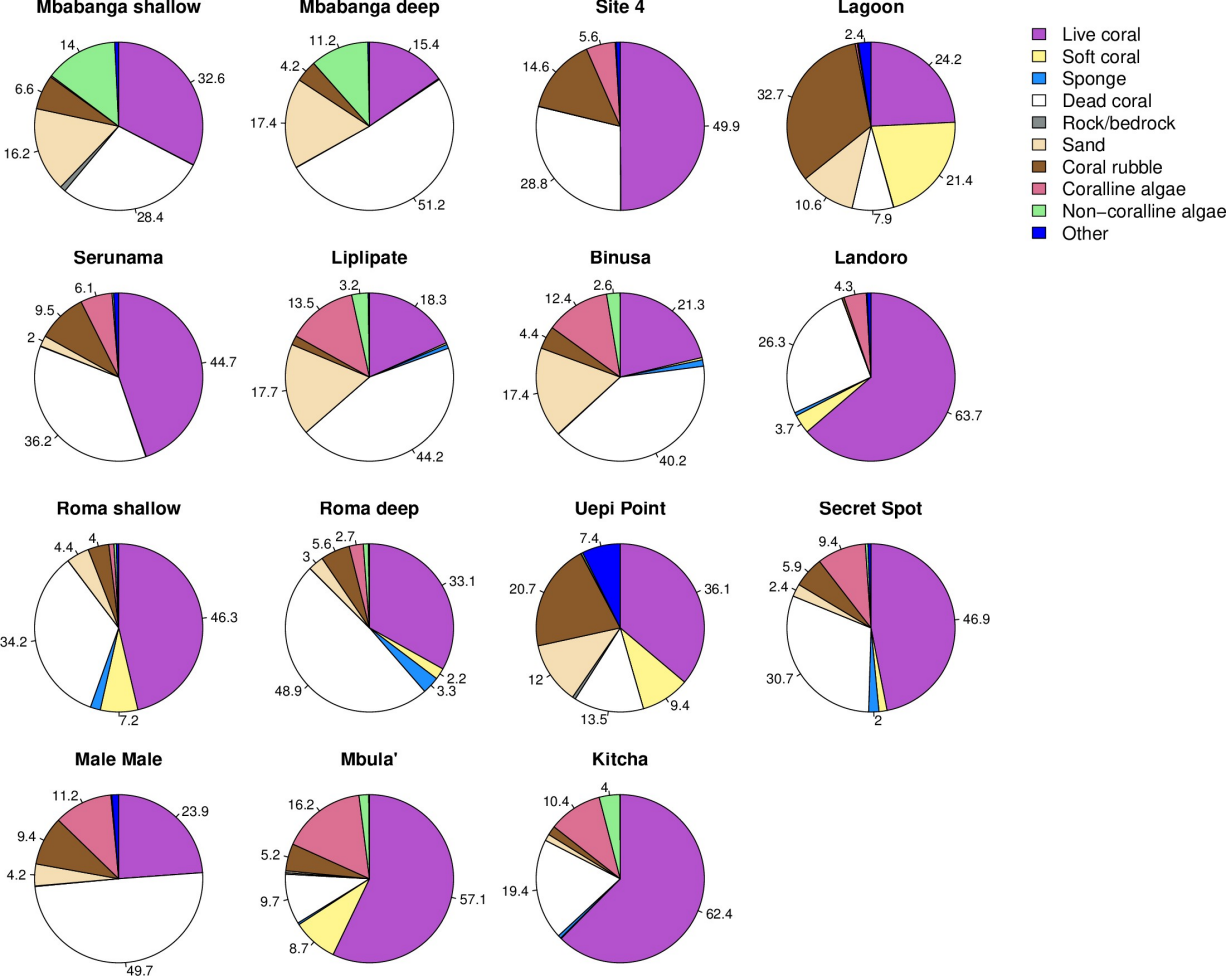
Substrate cover (%) at 13 sites spanning 4 islands in the Solomon Islands. Sites are arranged in order (top left to bottom right) of geographic location, from Mbabanga in the northwest to Kitcha in the southeast. Numbers are percentage of each substrate type with ≥ 2% cover.

**Fig 5:**
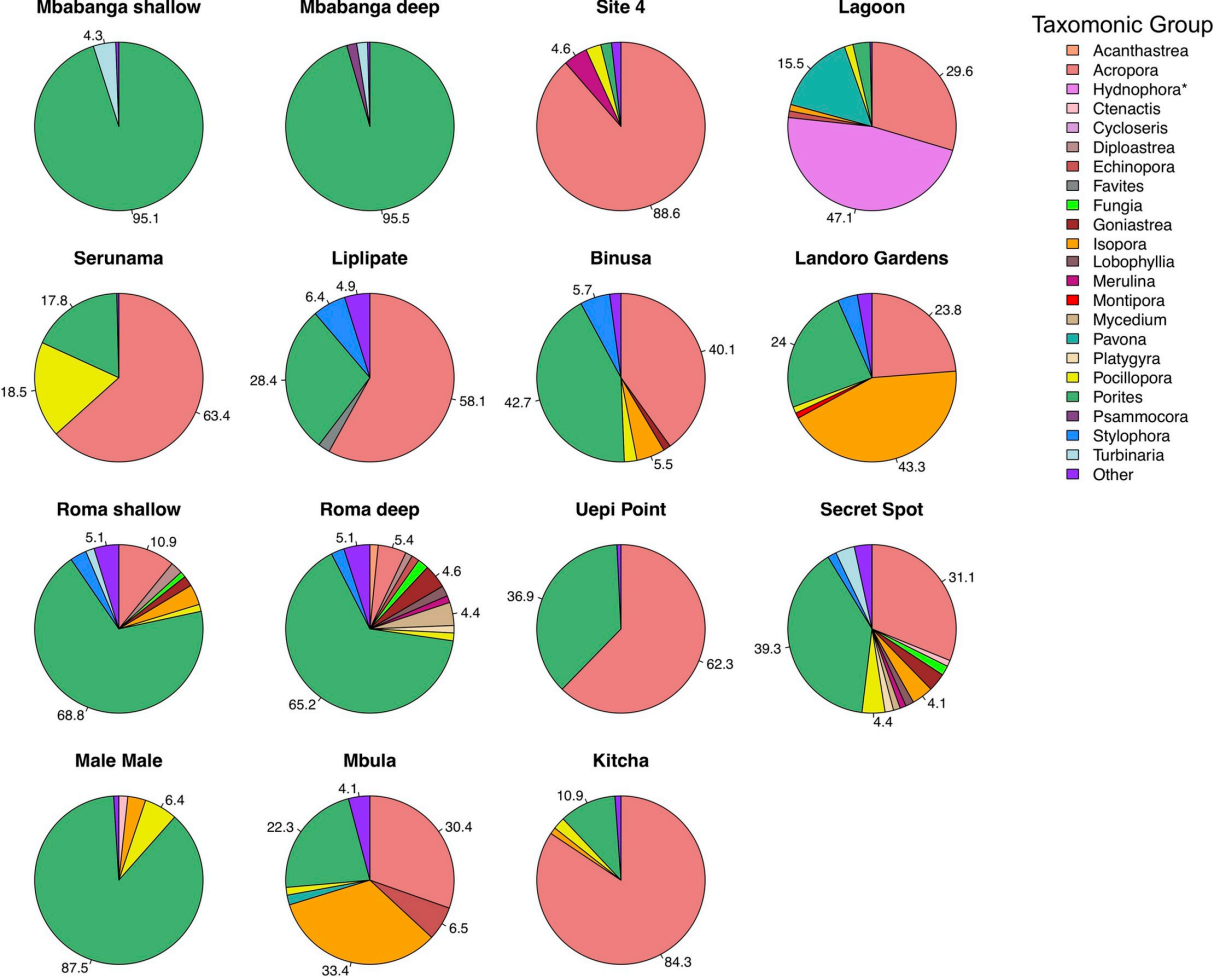
Coral taxonomic diversity (proportional abundance) at 13 sites spanning 4 islands in the Solomon Islands. Sites are arranged in order (top left to bottom right) of geographic location, from Mbabanga in the northwest to Kitcha in the southeast. Individual taxa making up ≤ 1% of the total coral are pooled as ‘Other’ (*n* = 17, S2 Data). Numbers are percentage of each coral taxon with > 4% proportional abundance. *No high-resolution context photos were taken of this coral; thus, we are less confident in our genus identification based on video transects alone.

**Fig 6:**
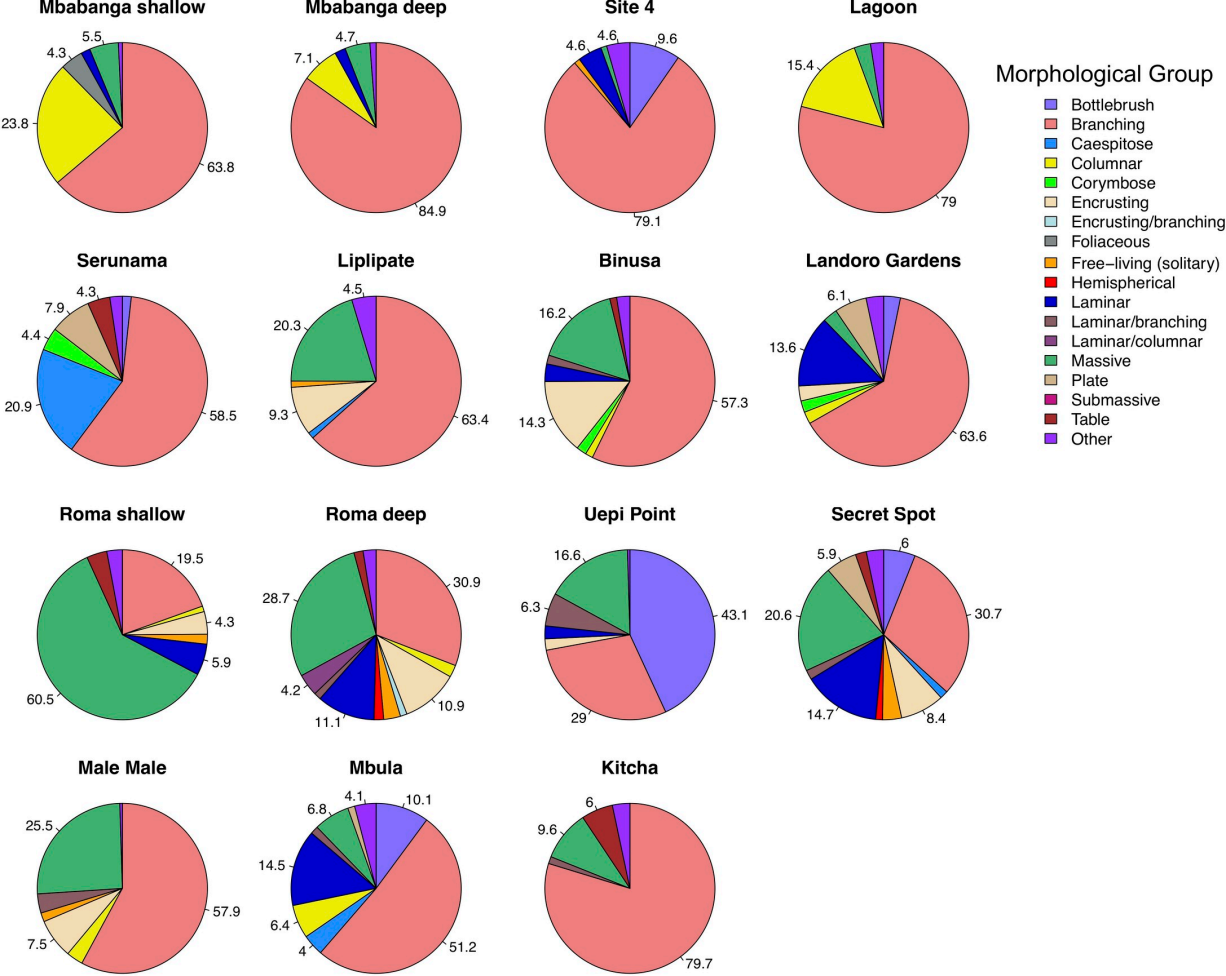
Coral morphological diversity (proportional abundance) at 13 spanning 4 islands in the Solomon Islands. Sites are arranged in order (top left to bottom right) of geographic location, from Mbabanga in the northwest to Kitcha in the southeast. Individual morphologies making up ≤ 1% of the total coral cover are pooled as ‘Other’ (*n* = 6, S2 Data). Numbers are percentage of each coral morphology with > 4% proportional abundance.

Hard coral cover ranged from 15.4 to 63.7% across the different sites (Fig 4). The highest percentage cover of hard coral occurred in Kitcha, Landoro Gardens, Mbula, and Site 4 and the lowest in Mbabanga deep, Liplipate, Binusa, and Male Male (Fig 4). In total, we identified 39 different genera across all sites, however, 17 genera made up ≤ 1% of the coral community at any given site (‘Other’ category in Figs 5 and 6). *Acropora* and *Porites* were the predominant genera in 12 of the 13 sites (Fig 5). Singly or combined, they accounted for 48% (Landoro Gardens) to 99% (Uepi Point) of coral community composition; *Isopora* was also a large component at 2 of these sites (Landoro Gardens, 43%; Mbula, 33%). At the remaining site (Lagoon), *Hydnophora* was the dominant genus (47%). Branching corals formed the most abundant morphological group at 11 of the sites, accounting for 31% (Secret Spot) to 85% (Mbabanga deep) of community composition (Fig 6). Bottlebrush (61%) and Massive (43%) corals were the dominant morphological groups at the remaining 2 sites (Uepi Point and Roma shallow, respectively).

**Fig 7:**
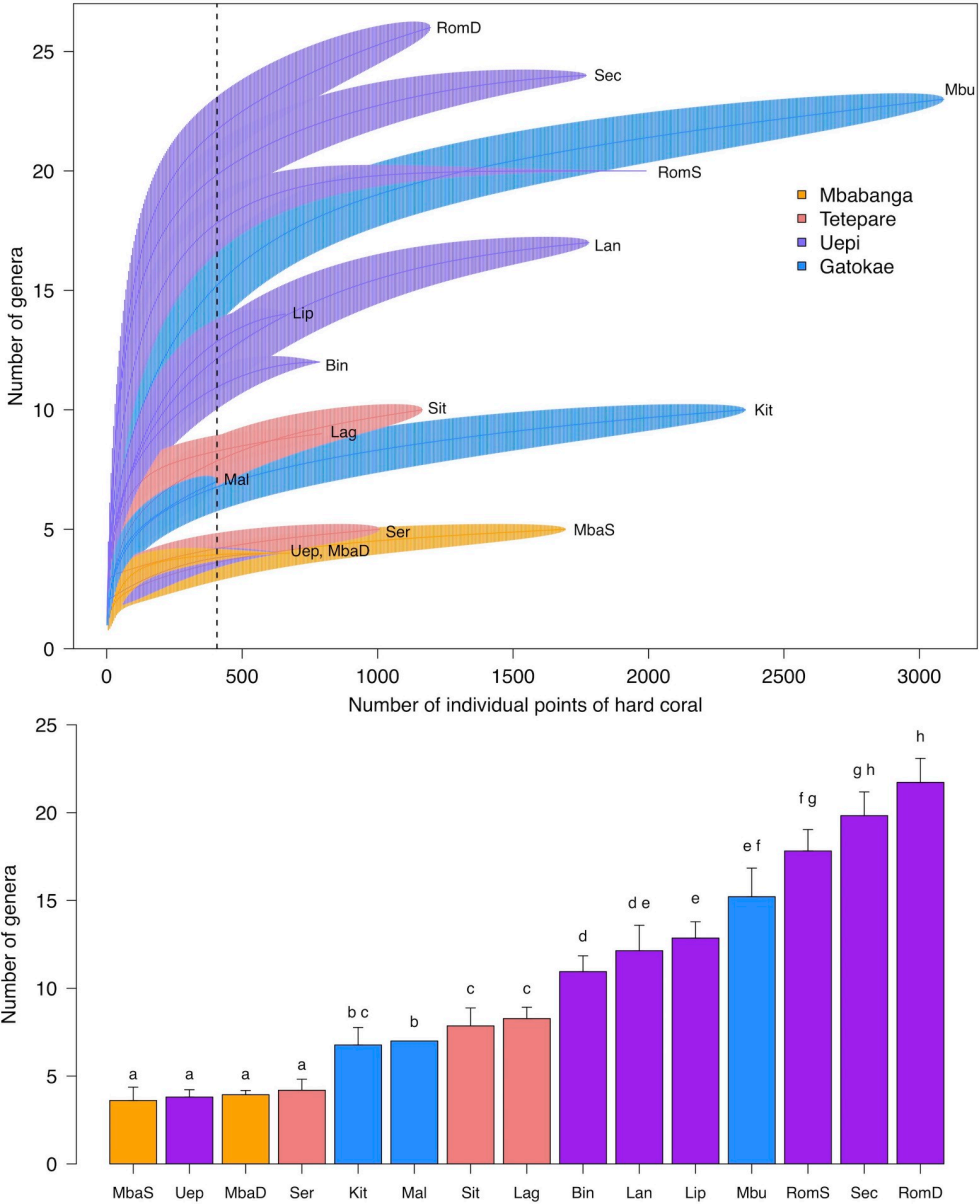
Coral taxonomic richness among 13 sites spanning 4 islands in the Solomon Islands. Top panel: sample-based rarefaction curves. The dotted line indicates the minimum number of points of live coral sampled across all sites (*n* = 407) used to compare taxonomic richness for a standardized number of individual points of live coral across sites. Bottom panel: number of genera (± 84% CI) from the standardized sample. Letters above bars indicate significant differences between sites at α < 0.05 (non-overlap of CI, [23]). See Fig 1 for site names.

**Fig 8:**
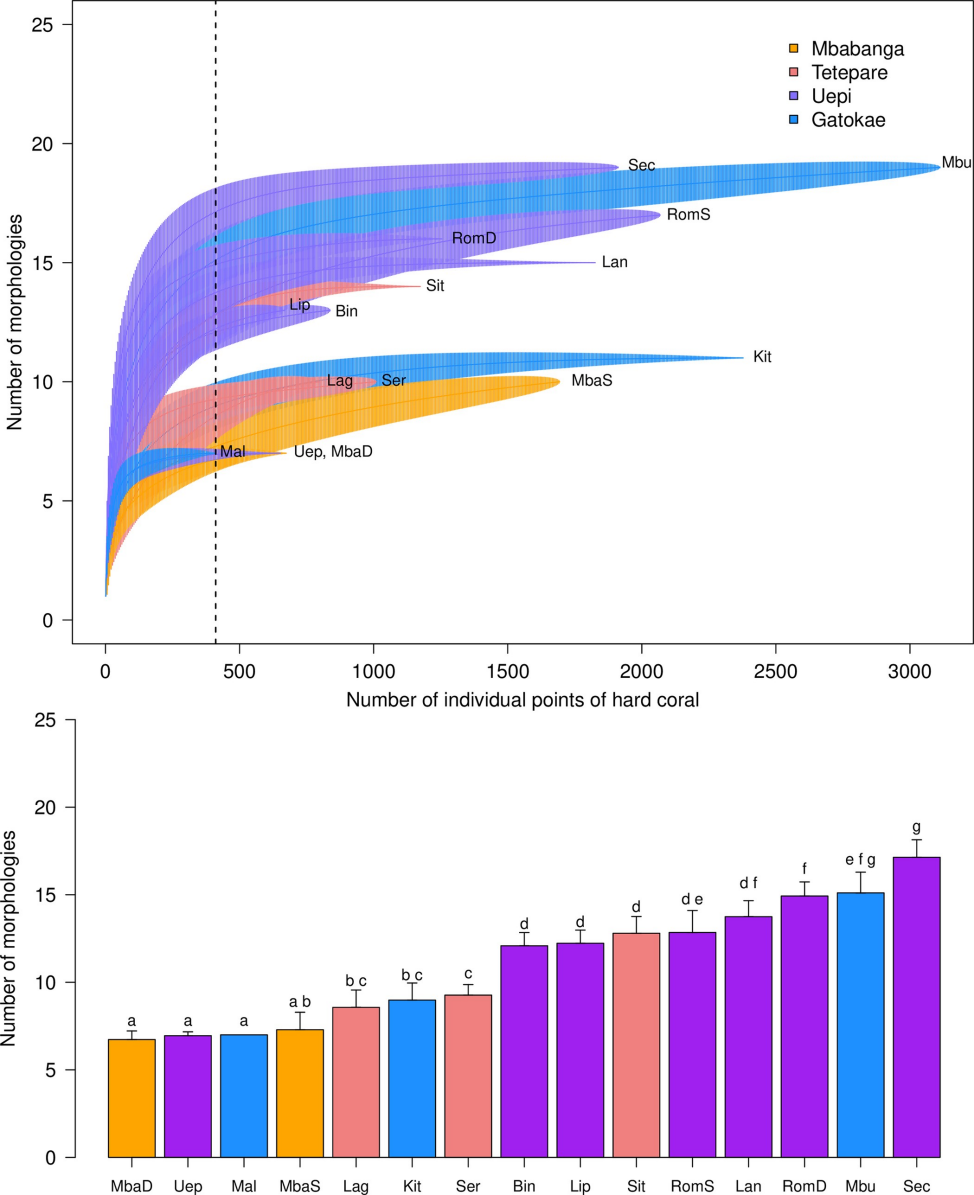
Coral morphological richness among 13 sites spanning 4 islands in the Solomon Islands. Top panel: sample-based rarefaction curves. The dotted line indicates the minimum number of points of live coral sampled across all sites (*n* = 411) used to compare morphological richness for a standardized number of individual points of live coral across sites. Bottom panel: number of morphologies (± 84% CI) from the standardized sample. Letters above bars indicate significant differences between sites at α < 0.05 (non-overlap of CI, [23]). See Fig 1 for site names.

**Fig 9:**
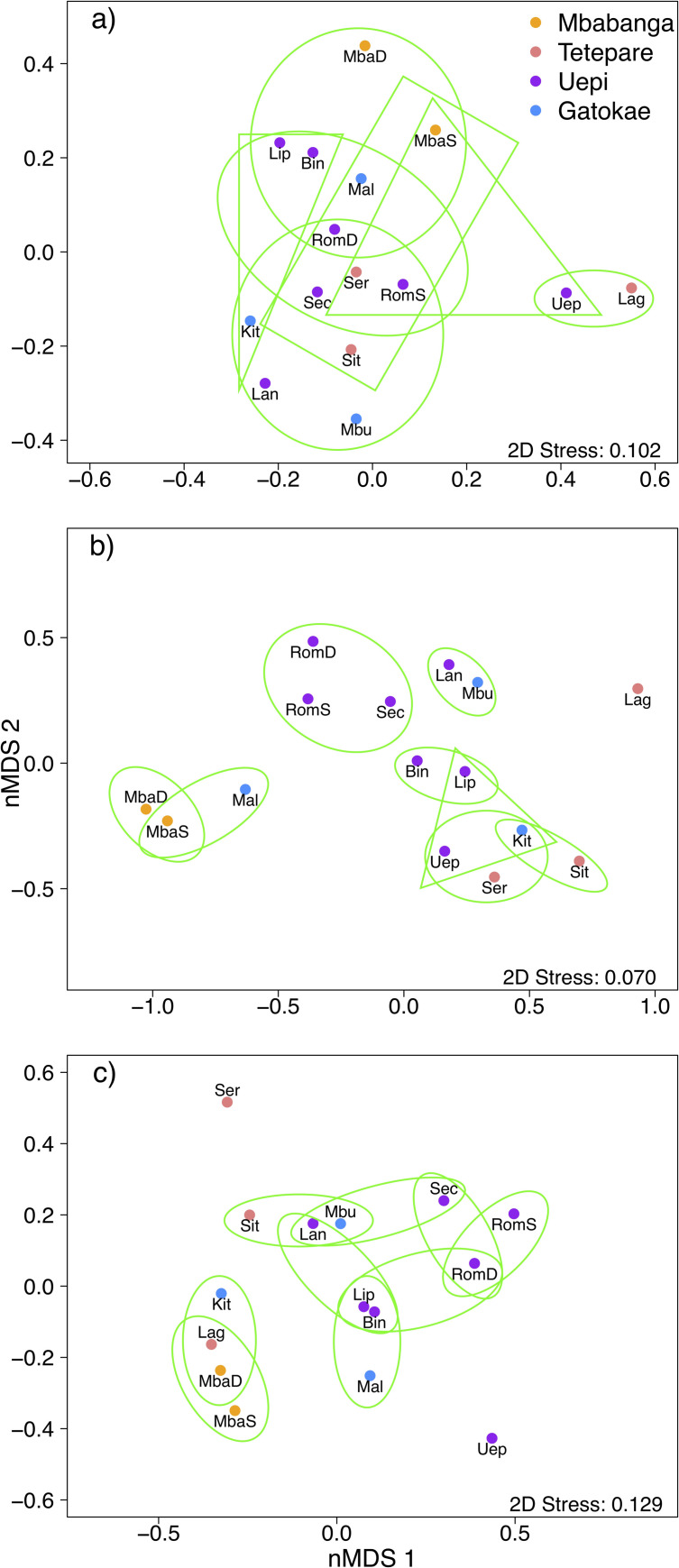
Differences in substrate cover, taxonomic and morphological diversity of coral assemblages among sites. Non-metric multidimensional scaling (nMDS) plots of (a) substrate cover (%) and (b) taxonomic and (c) morphological diversity (proportional abundance) of coral assemblages among 13 sites (and 2 depths for 2 sites: Mbabanga and Roma) across 4 islands (Mbabanga, Tetepare, Uepi, Gatokae) in the Western Province, Solomon Islands. Ellipses and triangles bound groups of sites of ≥ 70% similarity. Note the different axes scales. See Fig 1 for site abbreviations.

There were significant differences in both taxonomic and morphological richness among sites for the same sampling effort (Figs 7 and 8) with the composition of corals varying among sites (Fig 9). For the same sampling effort, generic richness ranged from 4 (Mbabanga shallow and deep, Uepi Point and Serunama) to 22 (Roma deep) genera out of 39 classifications across all sites, while morphological richness ranged from 7 (Mbabanga shallow and deep, Uepi Point, and Male Male) to 17 (Secret Spot) groups out of 23 categorized across all sites. Patterns in morphological richness among sites tended to reflect patterns in taxonomic richness. For example, Secret Spot had high taxonomic and morphological richness, while both depths at Mbabanga had among the lowest taxonomic and morphological richness (Figs 7 and 8). One exception was both depths at Roma, where taxonomic richness was higher than morphological richness (Figs 7 and 8).

Except for Mbula, sites with the highest coral cover were not among the most taxonomically or morphologically diverse. Sites with high coral cover consisted predominantly of branching *Acropora* (Site 4, Kitcha) or branching *Acropora* and *Isopora* (Landoro Gardens) (Figs 5 and 6). Sites with the lowest coral cover had low to intermediate taxonomic and morphological diversity and consisted predominantly of branching *Porites* (Mbabanga deep, Male Male, Binusa) or branching *Porites* and *Acropora* (Liplipate) (Figs 5 and 6).

Two sites of interest, where both the substrate composition and the coral assemblages differed markedly from the other sites, were Lagoon and Uepi Point (Fig 9). These sites were characterised by a comparatively high cover of coral rubble and soft coral (Fig 4), with the predominant hard coral consisting of branching *Acropora* and *Hydnophora* and columnar *Pavona* at Lagoon (Fig 3), and branching and bottlebrush *Acropora* and massive *Porites* at Uepi Point (Figs 5 and 6).

## Discussion

Despite the longest and most widespread global bleaching event to date in 2014–2017 [13], along with 3 severe (category 5) tropical cyclones (Ita, 2014; Pam, 2015; Donna, 2017) and various impacts of local pressures (Table 1) in the 4 years leading up to our study, the coral reefs that we surveyed in the Western Province, Solomon Islands were taxonomically and morphologically diverse. Rarefaction curves for the most diverse reefs surveyed suggest an asymptotic richness of upwards of 20–25 genera. For comparison, the generic richness of reef building corals for the entire Great Barrier Reef was estimated to be ~78 genera in 2001 [24]. Surveyed reefs also had a broad range of coral morphologies, including a high proportion of branching corals (20–85%), which tend to be more susceptible to bleaching-induced mortality [1]. Additionally, the range in cover of live hard coral at surveyed reefs (15–64%) was generally comparable to previous records from the region: 40–60% in 2003–2004 (Western Province [25]); 29–47% in 2004 (all provinces [26]); 20–38% in 2006–2007 (Western Province [27], includes some octocorals [blue coral, organ pipe coral] and hydrozoa [fire coral]); 21–60% in 2013 (Isabel Province [28]). In contrast, the northern Great Barrier Reef experienced 50% loss of live coral cover in 8 months during the 2016 bleaching event, with heavily bleached reefs undergoing a shift in coral assemblages from habitat forming branching and tabular species to species with less complex morphologies [29]. Similarly, 3 global bleaching events between 2011–2016, the most significant of which was in January 2016, resulted in a 44% decline in live coral cover in Indonesia’s Bali Barat National Park [30]. However, unlike the Great Barrier Reef, loss of coral cover in Indonesia was not accompanied by declines in taxonomic (genus) richness [30]. Reef-building capacity (percentage cover of reef building organisms: hard corals and coralline algae [31]), a proposed indicator of the resilience of reefs to large-scale disturbances, was also high across our study sites. Average percent cover of active reef builders in Western Province, Solomon Islands (44.6%) was comparable to that of remote uninhabited islands in the Central Pacific (45.2%), and substantially greater than inhabited islands (27.3%) in the same region [31]. Taken together, these results suggest that our surveyed reefs in this region remain healthy and functional in the face of recent global and local stressors.

The relatively healthy status of the reefs we surveyed likely reflects local variation in seawater temperature and bleaching stress. Reported concatenated temperatures across the greater Solomon Islands Bleaching Alert Area predicted Alert Level 1 almost annually between 2013 and 2018 [32]. However, at the scale of our individual sites, degree heating week only reached Coral Bleaching Alert Level 1 at a single site in a single year (Tetepare in 2016, S1 Fig). Interestingly, even at that site, coral did not show evidence of bleaching in 2018 and the reserve rangers reported that no bleaching had occurred in the previous 2 years. Local variation in seawater temperature can arise because of local hydrodynamics (e.g. stronger currents, eddy formation, upwelling of cold water, or greater mixing), and sea-surface temperature will not capture circulation patterns or the density structure of the water column below the mixed surface layer. For example, at Binusa, Uepi, extensive coral bleaching occurred in 2017 when subsurface upwelling of cold water, which normally occurs in June, was delayed resulting in prolonged heat stress at depth (Jill Kelly, *pers*. *comm*.*)*. Understanding local hydrodynamic processes can help identify potential refuges from bleaching. Greater than 10% live coral cover (the threshold for carbonate production) for all surveyed reefs, coupled with reduced exposure to bleaching-level stress during the 2014–2017 global bleaching event, suggests these reefs should be targeted for protective management strategies to maintain their functionality in response to increasing climate impacts [33].

There was evidence of a high magnitude disturbance event at Mbabanga, the site closest to the epicenter of the 2007 earthquake (Table 1). The deep reef at Mbabanga had the lowest cover of live coral (15%) that we recorded and the highest cover of dead coral (51%), while the shallow reef also had low coral cover (28%) and the highest cover of non-coralline algae (14%) that we recorded. Many of the dead colonies were overturned and broken up. Coral communities at both depths were characterized by low taxonomic and morphological diversity, consisting predominantly of branching *Porites* (Fig 3), a weedy life-history strategist often found colonizing recently disturbed habitats [34]. Interestingly, average hard coral cover at Mbabanga immediately following the 2007 earthquake was 18% and 29% at deep and shallow depths, respectively [35], indicating little recovery of hard coral at this site over the last decade.

Significant variation in coral cover and diversity across the 15 reefs surveyed, including among reefs on the same island, suggests that impacts of broad-scale (province- or island-wide) disturbance events are moderated at smaller scales by local environmental conditions. For example, Mbabanga (shallow and deep), Binusa, and Male Male were among the 4 sites with the lowest cover of hard coral even though they occurred on 3 different islands (Mbabanga, Uepi, and Gatokae). However, all 4 sites have a history of localized outbreaks of the corallivorous Crown-of-Thorns sea star *Acanthaster planci* (Table 1), likely contributing to the lower cover of live coral [36]. Enhanced predation on remaining corals by *A*. *planci* at Mbabanga following the 2007 earthquake may be inhibiting reef recovery [1]. Our findings are consistent with the notion that *A*. *planci* infestations are a major cause of damage to reefs in the Solomon Islands [26]. Asynchronies in coral cover and recovery trajectories also have been observed across small spatial scales (kilometers) on other Indo-Pacific reefs, providing further evidence of localized effects of regional to global disturbances [6].

Local-scale anthropogenic stressors for corals in the Solomon Islands include overfishing, nutrient run-off from agriculture, and sediment run-off from logging and mining [10]. High fishing pressure from nearby villages has negatively impacted the biomass of grazing fishes in Marovo Lagoon and the surrounding barrier islands, which include Uepi [37, 38]. Logging has been ongoing in the Solomon Islands since the 1970s, with 71% of the annual logging export coming from the Western Province [39]. Elevated concentrations of nutrients and suspended sediment are a permanent occurrence near river mouths of the large logged islands of Vangunu (near Uepi) and Gatokae, and coral cores show a significant increase in terrestrial inputs and sedimentation in the surrounding waters since 1995 [37]. Even under best management practices, current industrial-scale logging in the Solomon Islands is predicted to increase the total suspended sediment in rivers from a baseline annual average of 2.0 mg L-1 to 62 mg L-1 [40]. Despite proximal threats of overfishing and terrestrial inputs, reefs surveyed at Uepi and Gatokae in our study spanned a range of conditions, with different sites being among those with both the highest and lowest cover of hard coral and of taxonomic and morphological diversity. However, if local stressors persist or increase it could lead to a shift in coral communities towards more stress tolerant life histories, which would be reflected in declines in both taxonomic and morphological richness at these sites [34]. In comparison, sites at Tetepare, an established no-take marine protected area since 2003 and the only island in the Western Province that has not been logged, ranged from low (24%) to high (50%) coral cover and had consistently low taxonomic diversity and low to moderate morphological diversity.

The lack of a strong correlation between disturbance history and reef condition in our study may reflect the comparatively low anthropogenic impact on reefs in the Solomon Islands relative to more heavily populated areas, such as coastal Australia or Fiji. For example, although there is evidence to support perceived decreases in water quality in Marovo Lagoon by local inhabitants, the water quality in much of the lagoon is still excellent compared to global standards [37]. Additionally, although herbivore biomass at “heavily fished” reefs surrounding Vangunu and Gatokae Islands (20–150 kg ha-1 [37]) is below the threshold level above which fish herbivory affectively mediates competition between corals and algae (177 kg ha-1 [41]), increased numbers of smaller herbivorous fish coupled with low nutrient loads appear to be controlling algal biomass at these reefs [37]. In the absence of strong anthropogenic stressors, subregional variation in reef condition in the Solomon Islands may be driven by abiotic factors not explicitly measured in our study, such as local weather patterns, oceanographic conditions, or bathymetry. For example, although 2 of the 3 reefs surveyed on Tetepare occurred within a no-take LLMA, reefs on Tetepare were also the shallowest, and recovery of Indo-Pacific corals from bleaching is strongly related to reef depth regardless of whether reefs occur within or outside of no-take marine reserves [41]. Similarly, coral reef condition did not differ between protected and fished sites in Roviana Lagoon (New Georgia Island, Western Province), while adjacent unprotected barrier reef sites had significantly greater cover of live coral and less coral rubble [42]. This suggests that if global scale bleaching stress continues to increase, reefs such as the deeper ones that we surveyed (e.g. Uepi) may transition towards comparatively low taxonomic and morphological diversity, characteristic of Tetepare’s reefs, regardless of local management practices. Nonetheless, local conservation of reef fish assemblages and water quality may enhance the resilience of reefs to global stressors as the population of the Solomon Islands increases [43, 44], making effects of LLMAs on local-scale variation in reef health more apparent in the near future.

Alongside local environmental conditions, coral recruitment rate is one of the most important factors affecting the ability of reefs to recover from disturbance [45], and juvenile coral density is a strong predictor of recovery trajectory following severe bleaching events [41]. We observed coral recruits and juvenile corals on coral rubble at 4 sites (Site 4, Lagoon, Roma, and Liplipate), suggesting recovery at least at some sites in the region is not recruitment limited.

The few studies of coral reefs in the Solomon Islands to date suggest that high coral diversity, relatively healthy reefs and comparatively low exposure to common reef threats make the Solomon Islands a region of high coral conservation value [10, 26, 46]. Our survey of 13 sites in the Western Province supports this conclusion. Reef condition varied among sites, but overall, we found high cover of live coral, diverse coral assemblages and evidence of recovery capacity in the form of recent recruits and juvenile corals. Cooler sea surface temperatures recorded at our sites, relative to those concatenated for the region as a whole between 2013 and 2018, may have enabled corals to escape the recent global bleaching event. As coral reef conservation becomes increasingly urgent in the face of escalating cumulative threats, prioritising sites for management efforts is critical [45]. We propose that the Western Province, Solomon Islands be considered of high conservation priority based on: 1) the current status of the coral reefs surveyed in this study and their potential resilience to climate disturbances, 2) the high dependency of the local population on coral reef ecosystem services and 3) the potential for traditional governance structures to facilitate local stewardship of marine resources.

## Supporting information

S1 Fig Local-scale sea surface temperature and coral bleaching alerts. Sea surface temperature (SST, °C) and degree heating week (DHW, °C wk) for 4 islands (Mbabanga, Tetepare, Uepi, Gatokae) in the Western Province, Solomon Islands. Dotted orange lines at 4 and 8 DHW represent coral bleaching Alert Level 1 and 2, respectively. Data are from NOAA Coral Reef Watch (2018). NOAA Coral Reef Watch. 2018, updated daily. NOAA Coral Reef Watch Version 3.1 Daily Global 5-km Satellite Coral Bleaching Degree Heating Week Product, Jun. 3, 2013-Jun. 2, 2014. College Park, Maryland, USA: NOAA Coral Reef Watch. Data set accessed 2018-09-01 at https://coralreefwatch.noaa.gov/satellite/hdf/index.php.
(TIF)

S2 Fig Sample-based rarefaction curves for number of coral genera. Number of genera (± 95% CI) of coral with increasing number of video quadrats of approximately ~0.5 m2 for 13 sites (and 2 depths for 2 sites: Mbabanga and Roma) in the Western Province, Solomon Islands.
(TIF)

S1 Data Substrate composition. (XLSX)

S2 Data Taxonomic and morphological composition. (XLSX)
