# Supplementary figures and images for: Subregional variation in cover and diversity of hard coral (Scleractinia) in the Western Province, Solomon Islands following an unprecedented global bleaching event
Source: PLoS One. 2020 Nov 11;15(11):e0242153. doi: 10.1371/journal.pone.0242153 (PMC7657522; doi:10.1371/journal.pone.0242153)

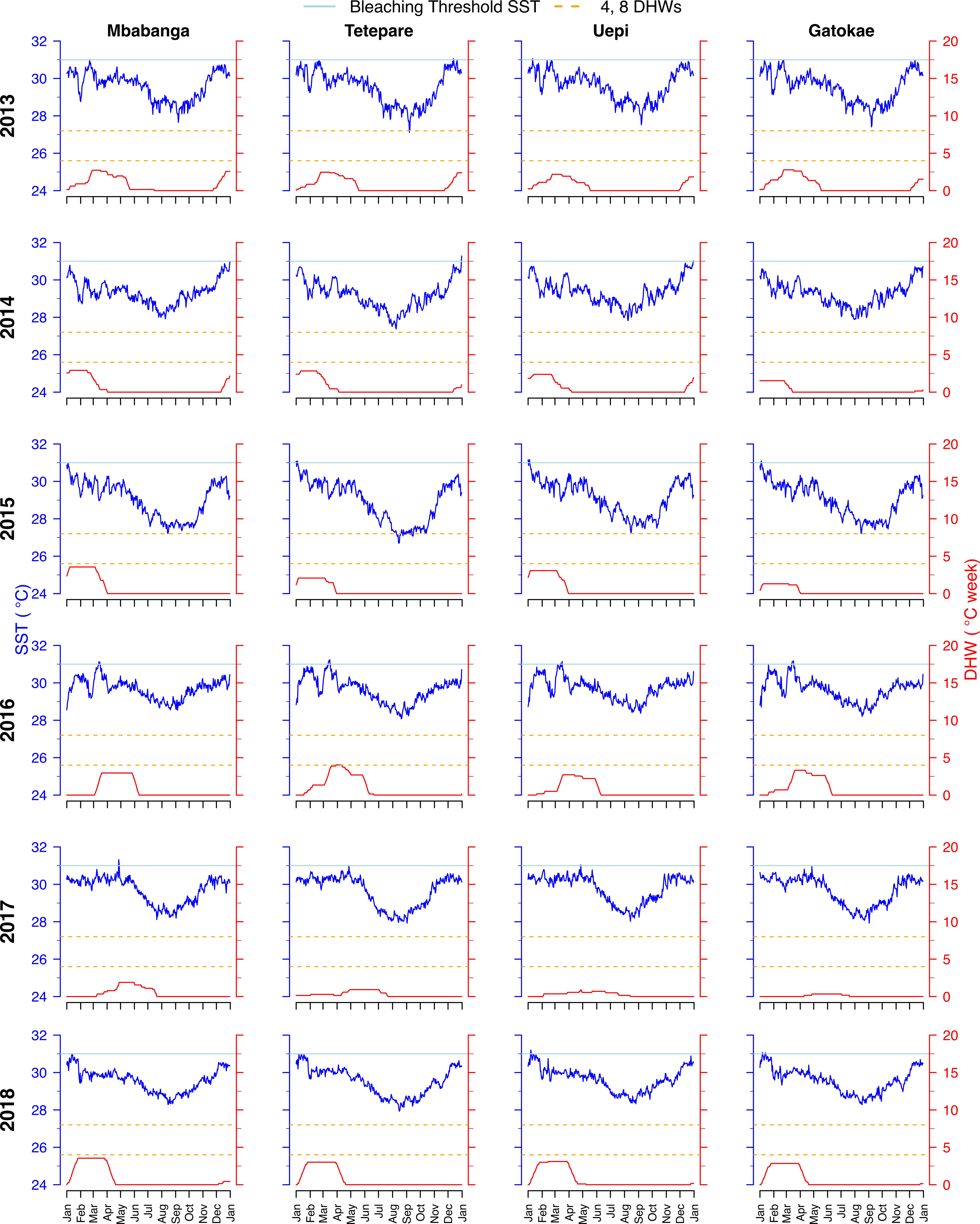

Supplement: S1 Fig — Sea surface temperature (SST, °C) and degree heating week (DHW, °C wk) for 4 islands (Mbabanga, Tetepare, Uepi, Gatokae) in the Western Province, Solomon Islands. Dotted orange lines at 4 and 8 DHW represent coral bleaching Alert Level 1 and 2, respectively. Data are from NOAA Coral Reef Watch (2018). NOAA Coral Reef Watch. 2018, updated daily. NOAA Coral Reef Watch Version 3.1 Daily Global 5-km Satellite Coral Bleaching Degree Heating Week Product, Jun. 3, 2013-Jun. 2, 2014. College Park, Maryland, USA: NOAA Coral Reef Watch. Data set accessed 2018-09-01 at https://coralreefwatch.noaa.gov/satellite/hdf/index.php. (TIF) [file pone.0242153.s001.tif]

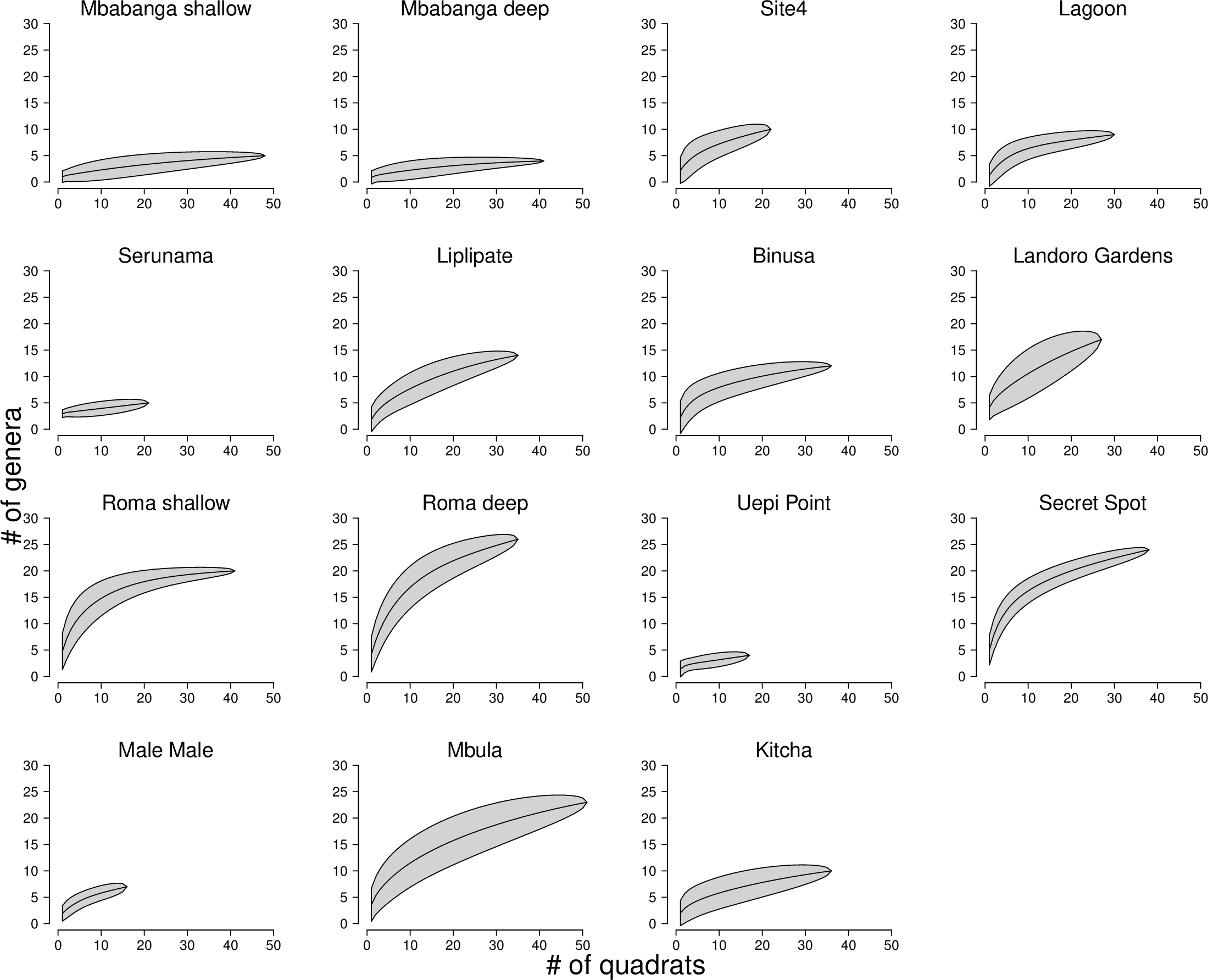

Supplement: S2 Fig — Number of genera (± 95% CI) of coral with increasing number of video quadrats of approximately ~0.5 m2 for 13 sites (and 2 depths for 2 sites: Mbabanga and Roma) in the Western Province, Solomon Islands. (TIF) [file pone.0242153.s002.tif]
